# Pancreatoduodenectomy without Vascular Resection in Patients with Primary Resectable Adenocarcinoma and Unilateral Venous Contact: A Matched Case Study

**DOI:** 10.1155/2018/1081494

**Published:** 2018-11-25

**Authors:** Luca Morelli, Raffaella Berchiolli, Simone Guadagni, Matteo Palmeri, Niccolò Furbetta, Desirée Gianardi, Matteo Bianchini, Niccola Funel, Giovanni Caprili, Luca Emanuele Pollina, Giulio Di Candio, Franco Mosca, Gregorio Di Franco, Alfred Cuschieri

**Affiliations:** ^1^General Surgery, Department of Surgery, Translational and New Technologies in Medicine, University of Pisa, Italy; ^2^EndoCAS (Center for Computer Assisted Surgery), University of Pisa, Italy; ^3^Vascular Surgery Unit, Department of Cardio Vascular Surgery, University of Pisa, Italy; ^4^Division of Surgical Pathology, University of Pisa, Italy; ^5^Institute for Medical Science and Technology, University of Dundee, Dundee, UK

## Abstract

**Purpose:**

To investigate the oncological outcome and survival of patients following a conservative approach on the portal-mesenteric axis, in an intraoperative ultrasound-selected group of pancreatoduodenectomy (PD), performed on patients with primary resectable with vascular contact (prVC) pancreatic ductal adenocarcinoma (PDAC).

**Methods:**

A consecutive series of patients who underwent PD for PDAC at our tertiary care center, between 2008 and 2017, were reviewed. A total of 156 PDs and 88 total pancreatectomies were performed during the study period, including 35 vascular resections. We identified a group of 40 (25.6%) patients with prVC-PDAC in whom after checking the feasibility with intraoperative ultrasound, we were able to perform PD by separation of the tumor from the portomesenteric axis avoiding vascular resection, without residual macroscopic disease (no vascular resection, nvrPD), and compared this group, using case-matched methodology, with the standard PD (sPD) group of primary resectable without vascular contact- (prwVC-) PDAC.

**Results:**

The median follow-up was 28.5 ± 23.2 months in the sPD group and 23.8 ± 20.8 months in the nvrPD group (*p* = 0.35). Isolated local recurrence rate was 2/40 (5%) in both groups. Additionally, there were no statistical differences in the systemic progression of the disease (42.5% sPD vs. 45% nvrPD, *p* = 0.82) or local plus synchronous systemic disease rates (2.5% sPD vs. 7.5% nvrPD, *p* = 0.30). The median survival was 22 months for the sPD group and 23 months for the nvrPD group, *p* = 0.86. The overall survival was similar in the two groups (1 y: 76.3% sPD vs. 70.0% nvrPD; 3 y: 35.6% vs. 31.6%; and 5 y: 28.5% vs. 25.3%; *p* = 0.80). *Conclusion*s. PD without vascular resection can be considered safe and oncologically acceptable in selected patients with preoperative diagnosis of prVC-PDAC. The poor prognosis of PDAC is related to the aggressive biology and systemic spread of the tumor, rather than the local control of the disease.

## 1. Introduction

Pancreatic ductal adenocarcinoma (PDAC) is one of the most biologically aggressive neoplasms characterized by poor prognosis with a mortality rate which currently ranks fifth in the list of cancer-related deaths. Surgical management remains the primary treatment, but resection is possible only in 15 to 20% of patients [1]. Approximately 65% of pancreatic tumors involve the pancreatic head. While patients with body or pancreatic tail cancer present usually with metastatic disease, lesions arising in the pancreatic head usually present with jaundice or symptoms related to duodenal obstruction. Consequently, patients with pancreatic head tumors may obtain improved survival from early diagnosis and treatment. In these cases, whenever possible, an R0 resection margin is associated with a 5-year survival rate of 20% in some reports. Because the pancreatic head is anatomically closely related to major arteries and veins, in 40% of patients, cancer involves one or more major vascular structures at the time of diagnosis [2]. The concept of borderline resectable pancreatic cancer [3] and the staging system proposed by the consensus statement of the Society of Abdominal Radiology and the American Pancreatic Association [4, 5] underlines the close relationship between the critical visceral vasculature and the local spread of the disease. The primary portal-mesenteric axis en bloc resection during pancreatoduodenectomy (PD) is considered a safe approach in experienced high-volume centers with acceptable morbidity and mortality and favourable prognosis compared to unresectable disease. However, the benefit in terms of improved disease-free survival by this aggressive major surgical resection remains unproven.

The present study was designed to investigate the oncological outcome (local/distant recurrence rate) and patients' survival after a conservative approach on the portal-mesenteric venous axis (PMA) in an intraoperative ultrasound- (IOUS-) selected group of PDs, in patients with primary resectable with vascular contact- (prVC-) PDAC.

## 2. Materials and Methods

From January 2008 to December 2017, 433 pancreatic resections were performed at the General Surgery Unit of the University of Pisa, of which a total of 156 were PDs for PDAC: 135 without vascular resection (VR) and 21 with VR. Eighty-eight patients underwent a total pancreatectomy, of which 51 for PDAC and 14 VR.

In this period, in our institution, primary pancreatic resection was contraindicated only when metastatic disease and invasion of the superior mesenteric or celiac artery were identified by preoperative imaging. PMA infiltration was not considered a contraindication for primary resection.

An intraoperative ultrasound (IOUS) with high-frequency probe (12-5 MHz, Intraoperative Biplane, BK Medical APS, Peabody, MA, USA) in order to check the relationship between the tumor and the PMA and SMA and to exclude occult liver metastasis was routinely performed in all patients undergoing PD. In case of the US sign of infiltration of PMA, we proceeded with an en bloc resection of the tumor with the tract of the vein involved, while for cases with a cleavage plane between the tumor and the PMA detected by ultrasound, we peeled off the vein from the tumor through a macroscopic tumor-free plane.

Preoperative workup included anamnestic collection, physical examination, blood exams, abdominal ultrasound (US), computed tomography (CT), magnetic resonance (MRI), and, depending on the case, other diagnostic methods, such as contrast-enhanced ultrasound (CEUS), endoscopic ultrasonography (EUS), and positron emission tomography (PET).

Preoperative biliary drainage was performed when bilirubin blood level was higher than 20 mg/dl. Patients underwent neoadjuvant therapy, when considered locally advanced at the diagnostic imaging.

Decisions about clinical management involved a multidisciplinary consultation between surgeons, oncologists, radiologists, and radiotherapists. Patients' data were stored in a dedicated institutional database.

For the present study, the grade of PMA invasion and the types of PDAC regarding tumor resectability (resectable, borderline resectable, locally advanced, and metastatic disease) were assigned retrospectively according to the National Comprehensive Cancer Network (NCCN) classification system [6] and to the International consensus on the definition and criteria of borderline resectable pancreatic ductal adenocarcinoma 2017 [7].

We identified a group of 40 patients with primary resectable disease with venous contact- (prVC-) PDAC, in whom, after checking the feasibility with IOUS, the vein had been peeled off the tumor without leaving macroscopic residual disease, avoiding a vascular resection (nvrPD group). We compared this group with a control group of 40 patients treated with a standard PD (sPD) for primary resectable without vascular contact- (prwVC-) PDAC. The control group was selected using a one-to-one case-matched methodology from the prospectively collected institutional database, where each patient of the nvrPD group was matched with a patient operated for prwVC-PDAC, using the following criteria: age, tumor size, grading, stage, and adjuvant therapy.

In these two groups, we evaluated differences in perioperative surgical results, local/distant recurrence, and survival. Prospectively collected details regarding pre-, intra-, and postoperative course and follow-up of patients of the two groups were retrospectively analysed and compared.

Preoperative and operative data included age, sex, BMI, ASA score, and operative time. Postoperative data included the length of hospital stay, postoperative morbidity (according to the Clavien-Dindo classification) [8], reintervention rate, and 30-day mortality. The medical complications recorded were pulmonary or urinary tract infections, cardiac complications, and neurological complications. The surgical complications included intra-abdominal fluid collections, surgical site infections, postoperative pancreatic fistula, digestive, and biliary or intraperitoneal hemorrhage. Postoperative pancreatic fistula (POPF) was defined and classified using the 2016-Revised International Study Group on Pancreatic Surgery classification (ISGPSc) [9]. Pathological data included the stage of pancreatic cancer, number of harvested lymph nodes, lymph node status, perineural space invasion, vascular space infiltration, vascular bed infiltration (medial pancreatic margin), and resection margin status.

Pathological data included the stage of pancreatic cancer according to the AJCC/TNM classification (8th edition) and the residual tumor classification (R0, free tumor margin > 1 mm; R1, free tumor margin if <1 mm or microscopic residual tumor; and R2; macroscopic residual tumor) [10, 11].

During the follow-up, data was collected on adjuvant therapy; local or systemic recurrence; the 1-, 3-, and 5-year overall survival (OS); and the disease-free survival (DFS) rate. The OS was defined as the length of time from the surgical resection of the pancreatic adenocarcinoma to the patient's death (last follow-up). Similarly, the DFS was defined as the time from the surgical resection to the diagnosis of recurrence (symptoms, radiological evidence, and/or pathological confirmation).

### 2.1. Statistical Analysis

For the data analysis, the *χ*^2^ test was used to define associations between categorical factors and surgical groups. Continuous variables with normal distribution are expressed as the mean ± standard deviation (SD) and compared using Student's *t*-test, with a significance being set at *p* < 0.05. Variables with an abnormal distribution are expressed as the median and compared using the Wilcoxon test. Survival was compared using the Kaplan–Meier curves and log-rank test. The statistical analysis was performed using SPSS (Statistical Production and Service Solution for Windows, SPSS Inc., Chicago, IL, USA) and STATA version 13 (STATA Corp., TX, USA).

## 3. Results

Demographic characteristics summarized in Table 1 showed no significant differences in clinical features between the two groups. The mean overall operative time (OT) was similar in both groups (435.9 ± 93.8 in the sPD group vs. 429.4 ± 86.1 in the nvrPD group, *p* = 0.75). Postoperative data (Table 2) showed no difference in the median length of postoperative stay between the two groups:15.0 days vs. 17.0 days for the sPD and nvrPD groups, respectively (*p* = 0.11). The overall postoperative complication rate was similar in both groups, occurring in 16 patients (40.0%) in the sPD group and in 22 patients (55.0%) in the nvrPD group (*p* = 0.18). Medical complications occurred in 10 cases (25%) and in 17 cases (42.5%) in the sPD and nvrPD groups, respectively (*p* = 0.10). Pancreatic fistula (according to the 2016-revised ISGPSc of POPF) developed in 3/40 patients (7.5%) in both groups: one grade B POPF (2.5%) and 2 (5%) grade C POPF in the sPD group and 3 (7.5%) grade C POPF in the nvrPD group; *p* = 0.55. Surgical complications occurred in 6 patients (15%) in the sPD group and in 5 patients (12.5%) in the nvrPD group (*p* = 0.75). The surgical complications in the sPD group were digestive hemorrhage (*n* = 2), bleeding associated with POPF grade C (*n* = 2), parietal hematoma (*n* = 1), and obstruction of the gastrointestinal anastomosis (*n* = 1). In the nvrPD group, surgical complications were digestive hemorrhage (*n* = 2) and bleeding associated with POPF grade C (*n* = 3). Digestive hemorrhage was treated with endoscopic intervention in all cases of both groups. Reintervention was required in 4 patients (10%) of the sPD group (2 grade C POPF, 1 parietal hematoma, and one gastrointestinal anastomosis obstruction) and in 3 patients (7.5%) of the nvrPD group (3 grade C POPF, *p* = 0.69). Postoperative mortality rate was 2.5% (1/40 cases) in both groups. Pathological findings are summarized in Table 3. No differences were reported between the two groups in terms of the stage of the disease, perineural and vascular bed infiltration. A mean of 28.4 ± 13.4 lymph nodes per patient was harvested in the sPD group versus 31.5 ± 14.1 in the nvrPD group (*p* = 0.32). The lymph nodes were positive in 29/40 cases (72.5%) in the sPD vs. 30/40 cases (75%) in the nvrPD (*p* = 0.80).

**Figure 1:**
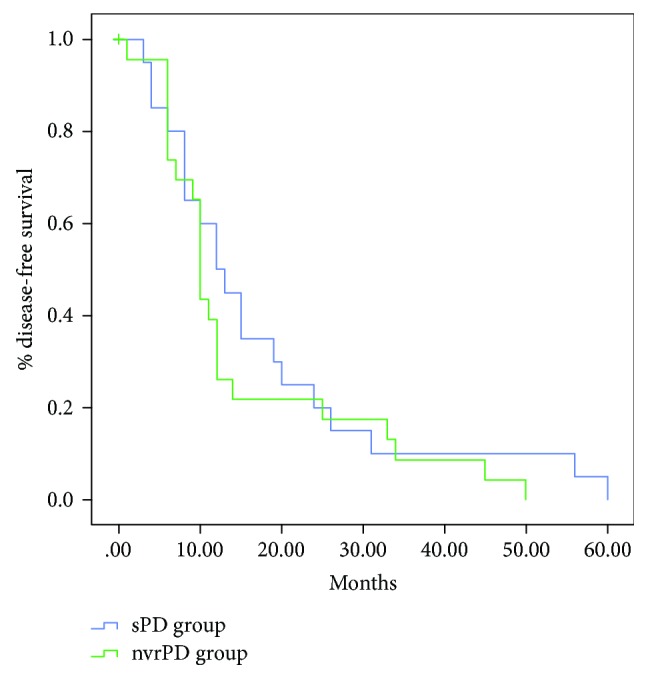
Disease-free survival (DFS) of the sPD and nvrPD groups.

**Figure 2:**
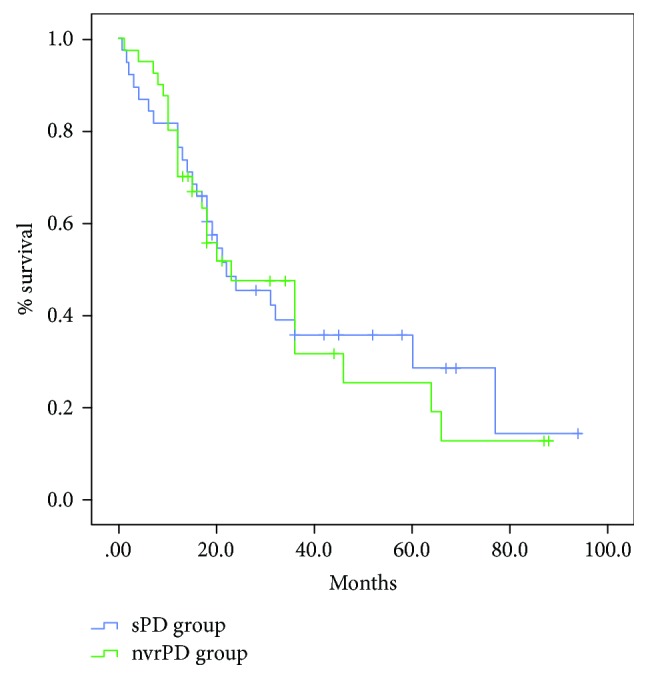
Overall survival (OS) of the sPD and nvrPD groups.

The median follow-up was 28.5 ± 23.2 months in the sPD group and 23.8 ± 20.8 months in the nvrPD group (*p* = 0.35). No patients in the two groups underwent neoadjuvant therapy because of primary resectable PDAC. In the sPD group, 21 patients (52.5%) vs. 20 patients (50%) in the nvrPD group received adjuvant therapy (*p* = 0.82). The disease-free survival (DFS) with the Kaplan–Meier method is shown in Figure 1. Isolated local recurrence was reported in 2/40 cases (5%) in both groups. No statistically significant difference in systemic progression or combined local and synchronous systemic disease rate was reported between the two groups: 17/40 cases (42.5%) in the sPD group vs. 18/40 case (45%) in the nvrPD group (*p* = 0.82) and 1/40 cases (2.5%) in the sPD group vs. 3/40 cases (7.5%) in the nvrPD group (*p* = 0.30, Table 4). Survival analysis using the Kaplan–Meier method is shown in Figure 2. The median survival was 22 months for the sPD group and 23 months for the nvrPD group (*p* = 0.80). No difference in the overall survival was observed between the two groups: 1-year overall survival rate was 76.3% in the sPD group vs. 70.0% in the nvrPD group; 3-year overall survival rate was 35.6% in the sPD group vs. 31.6% in the nvrPD group; and 5-year overall survival was 28.5% in the sPD group vs. 25.3% in the nvrPD group (*p* = 0.81).

## 4. Discussion

The pancreatic head is closely related to major arteries and veins, which are involved at the time of diagnosis in 40% of pancreatic ductal adenocarcinoma [2, 12]. According to the National Comprehensive Cancer Network (NCCN) guidelines, pancreatic cancer with superior mesenteric vein (SMV) and/or portal vein (PV) involvement greater than 180 degrees is considered borderline resectable unless the portal venous flow can be restored [3]. Several reports have shown similar short- and mid-term survival of patients with vein resection compared to patients treated with standard PD; specifically, Yekebas et al. [13] and Tseng et al. [14] did not report any difference in postoperative morbidity, mortality, and survival rate between the two groups. As a result of these reports, the surgical treatment in high-volume centers consists of mesenteric root mobilization and primary vascular resection en bloc, including cases of unilateral contact on preoperative imaging. The treatment includes all the true positive cases but overtreats many patients in whom histology of the excised specimen reveals only juxtavascular peritumoral inflammatory adherence rather than actual vascular infiltration [15]. Although feasible and safe, vascular resection is associated with increased perioperative risks, with increased morbidity associated with venous reconstruction [16, 17]. In patients with jaundice, portal clamping may further impair liver function [18]. The development of pancreatic fistula close to the suture line may promote thrombosis or pancreatic fistula-associated hemorrhage because the vascular resection and revascularization increase the likelihood of vessels being eroded by any pancreatic leak [19]. Furthermore, in patients treated with a total splenopancreatectomy, the compromised gastric venous drainage may necessitate a subtotal or total gastrectomy with increased postoperative morbidity [20]. Hence, some reports have raised doubts on the benefit of portal/mesenteric vein resection and have indicated that patients do not obtain any survival benefit after PV/SMV/PMA resection [14, 21, 22]. In fact, Roch et al. reported that vascular resection increased the 90-day mortality and that the vessel wall invasion does not represent a specific prognostic factor of overall survival because patients with and without vein resection obtained comparable overall survival rates at 1, 3, and 5 years. Indeed, the same authors report a higher rate of recurrence in the PMA resection group, probably because survival in these patients is largely due to aggressive tumor biology [23]. Zhou et al. in a meta-analysis including 19 studies found no differences in terms of overall survival between vascular and no vascular resection PDs. They also concluded that these patients' higher local recurrence did not materially contribute to poor survival, since this death was largely due to the systemic progression of the disease [24]. Preoperative staging can provide useful information on the relationship between the pancreatic tumor and the vascular wall to assist in planning the surgical resection. Multidetector computed tomography (MDCT) has provided the most accurate assessment of vascular involvement showing excellent sensitivity (100%) and specificity (72%) and high positive predictive value (89%) [3]. Al-Hawary et al. published a consensus statement of the Society of Abdominal Radiology and the American Pancreatic Association on preoperative cross-sectional imaging evaluation of tumor involvement of PMA [4]. The probability of vascular invasion is related to the contact of the tumor with the vessel. In a study by Springett and Hoffe, the probability of vascular invasion was increased to 40%, 80%, and 100% when the tumor had ≤180° contact, >180° contact, and 360°contact, respectively [25]. Moreover, venous caliber change at imaging is related with venous wall invasion as reported by Nakao et al. who demonstrated an absence of venous invasion on pathologic examination of resected specimens in patients without a radiographic appearance of vascular involvement while vascular invasion was revealed by pathologic examination in 51%, 74%, and 93% of patients with radiographic unilateral portal vein narrowing, bilateral portal vein narrowing, and complete portal vein obstruction with venous collateral formation, respectively [15]. Furthermore, the ability of CT to predict that vein resection is necessary approximates to 40% [26]. For these reasons, if the borderline resectable tumor with PMA invasion necessitates vascular resection to achieve a potentially curative resection, the focal contact between the neoplasm and the vascular wall is best considered a “black zone,” because at histopathological examination, the resected vessel is subsequently confirmed to be infiltrated by tumor in only 20-30% of cases [27]. In this respect, the severe desmoplastic reaction induced by pancreatic cancer contributes to the difficulty in the differentiation between adhesion and true vascular invasion. Thus, in this subgroup of patients, primary vascular resection may not be necessary. This difficulty in preoperative evaluation is related to the radiological definition of the vascular contact that differs from the pathological examination of the resected specimen.

The objective of the present study was to evaluate if the dissection of the tumor from the PMA in prVC could have similar surgical and oncologic outcome of a standard PD allowing surgeons to manage this situation without performing a vascular resection. In this single-center experience, a series of 40 prVC-PDAC were retrospectively compared with a comparable group of prwVC-PDAC, using a case-matched methodology, treated with standard PD. The use of IOUS proved to be very useful in the management of this prVC group as it enabled the definition of the limit of the PDAC and enabled differentiation between PMA involvements by the tumor from an inflammatory peritumoral process, enabling US-guided peeling with the preservation of PMA. The reported literature supports the usefulness of intraoperative ultrasound specially in identifying resectable head pancreatic lesions without infiltration of PMA [28, 29]. In fact, the adenocarcinoma appears as a hypoechogenic mass at IOUS, the margins and size of the lesion are more clearly visible and the relationship between the tumor and the critical vessels can be better evaluated.

Our study has demonstrated comparable oncologic results between the sPD and the nvrPD groups. Significantly, local recurrence was not higher in patients who had preservation of the vascular wall. Moreover, according to Zhou et al. who reported data that indicates that systemic progression is the leading cause of patients' death [24], we observed that the most important factor influencing long-term survival was the disease progression represented by peritoneal spread or hepatic metastasis. In fact, the patients' survival seems to be related to the aggressive biological behavior of the tumor, rather progressive local disease, although adequate resection to achieve local control remains important, but does not influence materially patient survival. In our opinion, the execution of a standard PD without vascular resection in some of these patients reduces the operative time, the length of hospital stays, and the morbidity without compromising clinical oncological outcome and overall survival.

The present study has some limitations, the first being related to its retrospective nature which inevitably raises possible selection bias. Secondly, the results of the prVC group could be overestimated as we did not include patients who underwent tangential or end-to-end vascular resection. However, the study underlines the importance of the intraoperative evaluation with US to specifically assess the extent of vascular and indeed the presence of vascular involvement to avoid the need for vascular resection during PD. In addition, to the best of our knowledge, this remains the first series comparing prwVC-PDAC and “no vascular resection” prVC-PDAC in a relatively large cohort, although we admit that further studies are needed to confirm the results of the present study.

## 5. Conclusion

PD without VR is a surgical approach that can be considered safe and oncologically acceptable in selected patients with preoperative prVC-PDAC. The poor prognosis of this disease is related to the aggressive biology and systemic spread of the tumor, rather than its local control. The present study does not support the need for vascular resection in all patients with prVC-PDAC.

## Figures and Tables

**Table 1:** Patient characteristics and operative data.

	sPD group (*n* = 40)	nvrPD group (*n* = 40)	*p* value
Mean age, year (range)	68.0 ± 11.1 (42-86)	68.8 ± 11.0 (46-92)	0.76
Male, *n* (%)	25 (62.5%)	23 (57.5%)	0.65
Female, *n* (%)	15 (37.5%)	27 (32.5%)	
Mean body mass index, kg/m^2^ (range)	25.0 ± 4.1 (16.1-39.0)	28.8 ± 5.0 (14.7-43.6)	0.99
ASA score, *n* (%)			0.49
ASA I	1 (2.5%)	0	
ASA II	10 (25%)	6 (15%)	
ASA III	24 (60%)	28 (70%)	
ASA IV	5 (12.5%)	6 (15%)	
Neoadjuvant therapy, *n* (%)	0	0	1
Mean overall operative time, min (range)	435.9 ± 93.8 (280-745)	429.4 ± 86.1 (300-700)	0.75

**Table 2:** Postoperative and pathological data.

	sPD group (*n* = 40)	nvrPD group (*n* = 40)	*p* value
Median length of postoperative stay, days [Q1–Q3]	15 (12-23)	17 (12-29.5)	0.11
Overall complications, number of patients (%)	16 (40%)	22 (55%)	0.18
Clavien-Dindo classification of postoperative complications			0.69
Grade I	2 (5%)	5 (12.5%)	
Grade II	7 (17.52%)	10 (25%)	
Grade III	4 (10%)	3 (7.5%)	
Grade IV	2 (5%)	3 (7.5%)	
Grade V	1 (2.5%)	1 (2.5%)	
Medical complications, *n* (%)	10 (25%)	17 (42.5%)	0.10
2016 ISGPS POPF, *n* (%)	3 (7.5%)	3 (7.5%)	1
Biochemical leak	2 (5%)	4 (10%)	0.59
Grade B POPF	1 (2.5%)	0	
Grade C POPF	2 (5%)	3 (7.5%)	
Surgical complications, *n* (%)	6 (15%)	5 (12.5%)	0.75
Reintervention, *n* (%)	4 (10%)	3 (7.5%)	0.69
Mortality	1 (2.5%)	1 (2.5%)	1

**Table 3:** Pathological data.

	sPD group (*n* = 82)	nvrPD group (*n* = 34)	*p* value
Stage, *n* (%)			0.79
Ia	1 (2.5%)	2 (5%)	
Ib	0	1 (2.5%)	
IIa	8 (20.0%)	7 (17.5%)	
IIb	29 (72.5%)	29 (72.5%)	
III	1 (2.5%)	2 (5%)	
Perineural space infiltration, *n* (%)	27 (67.5%)	33 (82.5%)	0.12
Vascular space infiltration, *n* (%)	12 (30%)	14 (35%)	0.63
Vascular bed infiltrations, *n* (%)	3 (7.5%)	5 (12.5%)	0.46
Mean lymph node harvest, *n* (range)	28.4 ± 13.4 (10-84)	31.5 ± 14.1 (9-69)	0.32
Patient with positive lymph nodes, *n* (%)	29 (72.5%)	30 (75%)	0.80
Grading, *n* (%)			0.55
G1	1 (2.5%)	2 (5%)	
G2	33 (82.5%)	29 (72.5%)	
G3	6 (15%)	9 (22.5%)	
R-status, *n* (%)			0.69
R0	37 (92.5%)	36 (90%)	
R1	3 (7.5%)	4 (10%)	

**Table 4:** Follow-up data.

	sPD group (*n* = 40)	nvrPD group (*n* = 40)	*p* value
Adjuvant therapy (*n*, %)	21 (52.5%)	20 (50%)	0.82
Isolated local recurrence (*n*, %)	2 (5%)	2 (5%)	1
Systemic progression (*n*, %)	17 (42.5%)	18 (45%)	0.82
Local and systemic disease (*n*, %)	1 (2.5%)	3 (7.5%)	0.30

## Data Availability

The data used to support the findings of this study are available from the corresponding author upon request.

## References

[1] Kim M., Kang T. W., Cha D. I. (2018). Prediction and clinical implications of portal vein/superior mesenteric vein invasion in patients with resected pancreatic head cancer: the significance of preoperative CT parameters. *Clinical Radiology*.

[2] Adham M., Mirza D. F., Chapuis F. (2006). Results of vascular resections during pancreatectomy from two European centres: an analysis of survival and disease-free survival explicative factors. *HPB*.

[3] Garces-Descovich A., Beker K., Jaramillo-Cardoso A., James Moser A., Mortele K. J. (2018). Applicability of current NCCN guidelines for pancreatic adenocarcinoma resectability: analysis and pitfalls. *Abdominal Radiology*.

[4] Al-Hawary M. M., Francis I. R., Chari S. T. (2014). Pancreatic ductal adenocarcinoma radiology reporting template: consensus statement of the society of abdominal radiology and the American Pancreatic Association. *Radiology*.

[5] Gilbert J. W., Wolpin B., Clancy T. (2017). Borderline resectable pancreatic cancer: conceptual evolution and current approach to image-based classification. *Annals of Oncology*.

[6] Tempero M. A., Arnoletti J. P., Behrman S. W. (2012). Pancreatic adenocarcinoma, version 2.2014: featured updates to the NCCN guidelines. *Journal of the National Comprehensive Cancer Network*.

[7] Isaji S., Mizuno S., Windsor J. A. (2018). International consensus on definition and criteria of borderline resectable pancreatic ductal adenocarcinoma 2017.

[8] Dindo D., Demartines N., Clavien P.-A. (2004). Classification of surgical complications: a new proposal with evaluation in a cohort of 6336 patients and results of a survey. *Annals of Surgery*.

[9] Bassi C., Marchegiani G., Dervenis C. (2017). The 2016 update of the International Study Group (ISGPS) definition and grading of postoperative pancreatic fistula: 11 years after. *Surgery*.

[10] Brierley J., Gospodarowicz M. K., Mary K., Christian W. C. TNM classification of malignant tumours. https://www.wiley.com/en-us/TNM+Classification+of+Malignant+Tumours%2C+8th+Edition-p-9781119263579.

[11] Gebauer F., Tachezy M., Vashist Y. K. (2015). Resection margin clearance in pancreatic cancer after implementation of the Leeds Pathology Protocol (LEEPP): clinically relevant or just academic?. *World Journal of Surgery*.

[12] Javed A. A., Bleich K., Bagante F. (2018). Pancreaticoduodenectomy with venous resection and reconstruction: current surgical techniques and associated postoperative imaging findings. *Abdominal Radiology*.

[13] Yekebas E. F., Bogoevski D., Cataldegirmen G. (2008). En bloc vascular resection for locally advanced pancreatic malignancies infiltrating major blood vessels. *Annals of Surgery*.

[14] Tseng J. F., Raut C. P., Lee J. E. (2004). Pancreaticoduodenectomy with vascular resection: margin status and survival duration. *Journal of Gastrointestinal Surgery*.

[15] Nakao A., Kanzaki A., Fujii T. (2012). Correlation between radiographic classification and pathological grade of portal vein wall invasion in pancreatic head cancer. *Annals of Surgery*.

[16] Tseng J. F. (2012). Proceed with caution: vascular resection at pancreaticoduodenectomy. *Annals of Surgical Oncology*.

[17] Castleberry A. W., White R. R., de la Fuente S. G. (2012). The impact of vascular resection on early postoperative outcomes after pancreaticoduodenectomy: an analysis of the American College of Surgeons National Surgical Quality Improvement Program database. *Annals of Surgical Oncology*.

[18] Yano K., Nishida M., Suzuki T. (1992). The effect of temporary hepatic artery or portal vein occlusion in obstructive jaundice. *Journal of Surgical Research*.

[19] Liang X., Shi L. G., Hao J. (2017). Risk factors and managements of hemorrhage associated with pancreatic fistula after pancreaticoduodenectomy. *Hepatobiliary & Pancreatic Diseases International*.

[20] Nakao A., Yamada S., Fujii T. (2018). Gastric venous congestion and bleeding in association with total pancreatectomy. *Journal of Hepato-Biliary-Pancreatic Sciences*.

[21] Kulemann B., Hoeppner J., Wittel U. (2015). Perioperative and long-term outcome after standard pancreaticoduodenectomy, additional portal vein and multivisceral resection for pancreatic head cancer. *Journal of Gastrointestinal Surgery*.

[22] Ravikumar R., Sabin C., Abu Hilal M. (2014). Portal vein resection in borderline resectable pancreatic cancer: a United Kingdom multicenter study. *Journal of the American College of Surgeons*.

[23] Roch A. M., House M. G., Cioffi J. (2016). Significance of portal vein invasion and extent of invasion in patients undergoing pancreatoduodenectomy for pancreatic adenocarcinoma. *Journal of Gastrointestinal Surgery*.

[24] Zhou Y., Zhang Z., Liu Y., Li B., Xu D. (2012). Pancreatectomy combined with superior mesenteric vein–portal vein resection for pancreatic cancer: a meta-analysis. *World Journal of Surgery*.

[25] Springett G. M., Hoffe S. E. (2008). Borderline resectable pancreatic cancer: on the edge of survival. *Cancer Control*.

[26] Tran Cao H. S., Balachandran A., Wang H. (2014). Radiographic tumor-vein interface as a predictor of intraoperative, pathologic, and oncologic outcomes in resectable and borderline resectable pancreatic cancer. *Journal of Gastrointestinal Surgery*.

[27] Zaky A. M., Wolfgang C. L., Weiss M. J., Javed A. A., Fishman E. K., Zaheer A. (2017). Tumor-vessel relationships in pancreatic ductal adenocarcinoma at multidetector CT: different classification systems and their influence on treatment planning. *Radiographics*.

[28] Bemelman W. A., de Wit L. T., van Delden O. M. (1995). Diagnostic laparoscopy combined with laparoscopic ultrasonography in staging of cancer of the pancreatic head region. *British Journal of Surgery*.

[29] Barabino M., Santambrogio R., Pisani Ceretti A., Scalzone R., Montorsi M., Opocher E. (2011). Is there still a role for laparoscopy combined with laparoscopic ultrasonography in the staging of pancreatic cancer?. *Surgical Endoscopy*.

